# Clinical characteristics and associated factors of airway allergic diseases among children: a cross-sectional study in a district of Beijing

**DOI:** 10.3389/fpubh.2026.1884452

**Published:** 2026-07-16

**Authors:** Li Sha, Siyu Ye, Tao Li, Shengchen Zhao, Mengyao Li, Changhao Xie, Weijing Wang, Xiaojun Zhan, Jieqiong Liang, Junting Liu, Qinglong Gu

**Affiliations:** 1 Department of Allergy, Capital Center for Children's Health, Capital Medical University, Capital Institute of Pediatrics, Beijing, China; 2 Allergy Center, Capital Center for Children's Health, Capital Medical University, Beijing, China; 3 Department of Otolaryngology-Head and Neck Surgery, Capital Center for Children's Health, Capital Medical University, Capital Institute of Pediatrics, Beijing, China; 4 Child Health Big Data Research Center, Capital Center for Children's Health, Capital Medical University, Capital Institute of Pediatrics, Beijing, China; 5 Graduate School of Peking Union Medical College, Beijing, China

**Keywords:** airway allergic diseases, allergic rhinitis, asthma, children, lifestyle

## Abstract

**Introduction:**

This study aims to investigate the impact of genetic factors, dietary habits, and social behavior patterns on allergic airway diseases among children in Beijing, providing evidence for disease prevention and early intervention.

**Methods:**

A random cluster sampling method was employed to survey 2,132 children aged 6–12 years in a district of Beijing. Participants were categorized into disease and control groups according to ARIA and GINA guidelines. Data on genetic background, dietary habits, and social behavior patterns were collected via questionnaires and physical examinations. Results were presented using descriptive statistics, with associated factors analyzed through binary logistic regression.

**Results:**

Among 2,132 children, the prevalence of allergic airway disease was 28.8%. Compared with the control group, the disease group exhibited significantly higher proportions of males (*P* < 0.001), parents with higher education (*P* = 0.020, *P* = 0.011), household annual income exceeding 120,000 yuan (*P* = 0.007), exposure to second-hand smoke (*P* = 0.030), pet ownership (*P* = 0.003), and personal/familial atopic history (*P* < 0.001). They also reported higher consumption of meat (*P* = 0.037) and soft drinks (*P* = 0.020). Their depression (*P* < 0.001) and sleep (*P* < 0.001) questionnaire scores were significantly higher. Compared to children with allergic rhinitis without asthma, those with asthma exhibited higher rates of multiple allergy-related histories, longer close-distance screen exposure times, and higher sleep questionnaire scores than children with allergic rhinitis alone (*P* < 0.05). Univariate analysis identified male gender (*P* < 0.001, aOR, 1.644; 95% CI: 1.302 to 2.076), a history of asthma in first-degree relatives (*P* = 0.040, aOR, 3.544; 95% CI: 1.059 to 11.933), a self-reported history of food allergies (*P* < 0.001, aOR, 2.574; 95% CI: 1.691 to 3.916), and a history of atopic dermatitis (*P* < 0.001, aOR, 7.224; 95% CI: 3.087 to 16.903), and frequency of meat consumption (*P* = 0.043, aOR, 1.290; 95% CI: 1.008 to 1.651) were identified as independent associated factors with statistically significant differences.

**Discussion:**

This study investigated the impact of various factors on the prevalence of allergic airway diseases among children in a district of Beijing. In addition to revealing associations between allergic airway diseases and gender, genetic factors, tobacco exposure, and a history of atopic dermatitis, the study also found that meat consumption is an independent associated factor for allergic airway diseases in children. Furthermore, we found a certain association between localized allergic airway inflammation and sleep disturbances as well as depressive symptoms. Our findings underscore the need for public health interventions that simultaneously address multiple lifestyle factors to reduce the risk of allergic diseases.

## 1 Introduction

Airway allergic diseases represent a group of **heterogeneous conditions** characterized by non-specific chronic airway inflammation and airway hyper-responsiveness, typically triggered by inhaled allergens. The clinical manifestations primarily include **allergic rhinitis**, which presents as rhinorrhea, sneezing, nasal itching, and congestion. When the lower airway is involved, symptoms such as chest tightness, dyspnea, cough, and wheezing may occur, and severe cases, including which with at least one of the following manifestations: poorly controlled, frequent severe attacks, severe attacks, or airflow limitation, (1) can progress to life-threatening respiratory failure.

Airway allergic diseases are a significant burden on global health. Current data indicate that approximately 400 million people worldwide suffer from allergic rhinitis and 300 million are affected by bronchial asthma (2). In China, the incidence of these diseases is increasing annually, with a trend toward onset at younger ages. Epidemiological data from 2010 showed that the prevalence of asthma among urban children aged 0–14 years is 3.02%, affecting approximately 30 million children nationwide (3). Furthermore, the overall prevalence of allergic rhinitis among Chinese children and adolescents from 2001 to 2021 is estimated to be 18.46% (4).

These diseases not only impair quality of life, but can also be life-threatening, imposing a substantial burden on individuals, families, and healthcare systems. The rapid rise in prevalence is associated with environmental and climatic shifts as well as changes in dietary and social behavior patterns. However, research in these specific areas remains limited. Therefore, this study investigated the influence of genetic factors, diet, and social behavior on the prevalence of airway allergic diseases among school-aged children in a district of Beijing, providing a scientific basis for disease prevention and early intervention.

## 2 Materials and methods

### 2.1 Study subjects

This was a observational cross-sectional study. Using a random cluster sampling method, 2,132 school-aged children (aged 6–12 years) from seven primary schools in the Tongzhou District of Beijing were surveyed from May to July in 2023. All participants completed a questionnaire and underwent a physical examination. Participants were categorized into the **disease (case) group** or the **control group** based on the presence of airway allergic diseases. Diagnostic criteria for allergic rhinitis and asthma followed the **ARIA** and **GINA** guidelines, respectively (5, 6). The study was approved by the Ethics Committee of the Capital Institute of Pediatrics (Approval No.: SHERLL2022043).

### 2.2 Research methods and content

The assessment consisted of a questionnaire survey and a physical examination.

#### 2.2.1 Questionnaire survey

The questionnaire included the following:

Demographic and family background: Parents' age, education level, and average annual family income (categorized as > or ≤ 120,000 RMB).

Environmental factors: Secondhand smoke exposure (defined as >15 min in the past week) and pet ownership.

Atopic History: Personal and family history of allergic rhinitis, asthma, food allergies, and atopic dermatitis. Regarding allergy history, we compiled data from formal diagnoses made by clinicians and self-reports from parents. For children reported by parents to have allergic rhinitis and asthma, we used the International Study of Asthma and Allergy in Children (ISAAC) questionnaire for confirmation; this questionnaire is commonly used for preschool and school-aged children and has demonstrated good reliability and validity among school-aged children in China (7, 8). For children reported by parents as having atopic dermatitis, we used the Hanifin and Rajka diagnostic criteria, which are widely recognized and considered the gold standard for diagnosing atopic dermatitis (9, 10). Regarding food allergies, we recorded a history of specific food allergies to milk, eggs, fish and shellfish, peanuts, tree nuts, wheat, soy products, fruits, and vegetables; all of these food allergies were self-reported by parents.

For atypical asthma, our diagnostic criteria are as follows: Cough-variant asthma must meet the following four criteria: 1. A cough lasting >4 weeks, often occurring or worsening during exercise, at night, and/or in the early morning, primarily a dry cough without wheezing; 2. No clinical signs of infection, or failure to respond to prolonged antibiotic treatment; 3. Diagnostic treatment with anti-asthma medications is effective; 4. Chronic cough caused by other factors has been ruled out.

Early life factors: Delivery mode and feeding methods within the first 6 months.

Physical activity and lifestyle: Weekly exercise frequency, daily exercise duration, and extracurricular screen time.

Dietary habits: Consumption frequency of meat, eggs, dairy, high-calorie foods, sugary beverages, and snacks.

Sleep quality: We used the OSA-specific quality of life for children with obstructive sleep apnea 18 items survey (OSA-18) and the Chinese version of the Children's Sleep Habit Questionnaire (CSHQ). These self-assessment scales have been validated for use with Chinese-speaking populations (11, 12). The OSA-18 scale consists of five dimensions: sleep disturbances, physical symptoms, low mood, daytime functioning, and the extent of impact on the child's caregiver. Each dimension is further subdivided into three to four items. The frequency of symptoms is recorded on a seven-point scale: 1 = None, 2 = Almost never (0–1 times per month), 3 = Very rarely (2–3 times per month), 4 = Sometimes (1–2 times per week), 5 = Often (3 times per week), 6 = Most of the time (every other day), 7 = Every day. The total score ranges from 18 to 126; a higher score indicates a more severe impact on quality of life. A score < 60 indicates mild severity, 60–80 indicates moderate severity, and >80 indicates severe severity (13). The CSHQ consists of 33 items across eight subscales (resistance to bedtime, delay in falling asleep, sleep duration, sleep anxiety, nighttime awakenings, parasomnias, sleep-related breathing disorders, and daytime sleepiness). It uses a three-point scale to assess the average weekly frequency of sleep-related symptoms: Usually (5–7 times/week), Sometimes (2–4 times/week), and Rarely (0–1 time/week). The cutoff score is 41; according to this scale, a higher score indicates poorer sleep quality (14).

Depressive Symptoms: We used the Depression Self-Rating Scale for Children (DSRS-C). This self-report instrument has been modified and validated for use among Chinese-speaking populations (15). This questionnaire, developed by Birleson, is an 18-item self-report scale used to screen children for depression. It asks children to rate their own condition over the past week on a three-point scale. Responses are scored on a scale of 0 to 2, with a total score of 36. The cutoff score is set at 15; higher scores indicate relatively severe depression (16).

#### 2.2.2 Physical examination

Height (cm) and weight (kg) were measured to calculate the **Body Mass Index (BMI)**.

### 2.3 Statistical methods

Data analysis was performed using the SPSS Statistics statistical program version 30.0 for Windows 10 (IBM Corp., Armonk, New York, United States). Normally distributed quantitative data are presented as the mean ± standard deviation (x±s) and were compared using independent sample *t*-tests. Non-normally distributed data are presented as median (interquartile range) [M (P25, P75)] and were compared using the Mann-Whitney *U* test. Categorical data are presented as counts and percentages (%) and were analyzed using Pearson's chi-square test. Associated factors were identified using binary logistic regression analysis. Statistical significance was set at *P*-value < 0.05.

## 3 Results

### 3.1 Demographic characteristics

Of the 2,132 children, 614 were classified as having airway allergic diseases (disease group), and 1,518 were classified as controls. In the disease group, 371 (60.4%) patients were male and 243 (39.6%) were female. The median age was 9 (8, 10) years, and the median BMI was 17.6 (15.5, 21.8) kg/m^2^. In the control group, 768 (50.6%) were boys and 750 (49.4%) were girls; median age was 9 (8, 10) years and median BMI was 18.2 (15.8, 22.8) kg/m^2^. Age and BMI did not differ significantly between the groups, whereas the proportion of boys was higher in the disease group (*P* < 0.001).

### 3.2 Comparison between the airway allergy and control groups in terms of basic family conditions, personal and family history of atopic diseases, birth and feeding conditions, and dietary status

Compared with the controls, the disease group had a higher proportion of fathers and mothers with higher education (*P* = 0.020 and *P* = 0.011, respectively) and a higher proportion of households with an annual income >120,000 yuan (*P* = 0.007). Parental age at birth was lower in the disease group (paternal, *P* = 0.002; maternal, *P* = 0.009). Regarding environmental factors, secondhand smoke exposure (*P* = 0.030) and pet ownership (*P* = 0.003) were more common in the diseased group. With respect to atopic history, the disease group had a higher proportion of a family history of allergies (*P* < 0.001), asthma and other allergic diseases in first-degree relatives (both *P* < 0.001), asthma in second-degree relatives (*P* = 0.037), other allergies in second-degree relatives (*P* < 0.001), self-reported food allergies (*P* < 0.001), and atopic dermatitis (*P* < 0.001). In the dietary assessment, the disease group reported more frequent meat consumption (*P* = 0.037) and soft drink consumption (*P* = 0.020), while the other dietary variables did not differ significantly (all *P* > 0.05; Table 1).

**Table 1:** Comparison of basic family conditions, personal and family history of atopic diseases, birth and feeding conditions, and dietary status between the airway allergy group and the control group.

Variables	Airway allergic diseases (*n* = 614)	Control (*n* = 1518)	t/Z/χ^2^	*P* value
Sex (male), *n* (%)	371 (60.4%)	768 (50.6%)	16.979	**< 0.001**
Age	9 (8, 10)	9 (8, 10)	−0.875	0.382
BMI (kg/m^2^)	17.6 (15.5, 21.8)	18.2 (15.8, 22.8)	−1.953	0.051
Father's age at birth of child	29 (27, 32)	30 (27, 34)	−3.107	**0.002**
Mother's age at birth of child	28 (26, 30)	29 (26, 32)	−2.599	**0.009**
Alcohol consumption				
Never	596 (99.8%)	1,452 (99.9%)	0.026	0.872
More than once	1 (0.2%)	2 (0.1%)		
Second-hand smoke exposure				
Never	397 (64.7%)	1,033 (68.1%)	2.321	**0.020**
More than once	203 (33.1%)	415 (27.3%)		
Father's highest educational attainment				
Below bachelor's degree	291 (48.3%)	811 (53.9%)	5.398	**0.020**
Bachelor's degree or above	312 (51.7%)	695 (46.1%)		
Mother's highest educational attainment				
Below bachelor's degree	267 (44.6%)	762 (50.7%)	6.429	**0.011**
Bachelor's degree or above	332 (55.4%)	741 (49.3%)		
Average annual household income (yuan)				
< 120,000	251 (40.9%)	718 (47.3%)	7.267	**0.007**
≥120,000	363 (59.1%)	800 (52.7%)		
Feeding methods before 6 months of age				
Breastfeeding	343 (56.5%)	910 (61.5%)	5.936	0.051
Formula feeding	88 (14.5%)	167 (11.3%)		
Combined feeding	176 (29.0%)	402 (27.2%)		
Family history of allergies				
Yes	148 (25.2%)	120 (8.3%)	103.874	**< 0.001**
No	440 (74.8%)	1,325 (91.7%)		
First-degree relative with asthma				
Yes	15 (2.6%)	9 (0.6%)	13.444	**< 0.001**
No	599 (97.4%)	1,509(99.4%)		
First-degree relatives with other allergies				
Yes	109 (18.6%)	83 (5.7%)	80.348	**< 0.001**
No	478 (81.4%)	1,363 (94.3%)		
Second-degree relative with a history of asthma				
Yes	16 (2.7%)	20 (1.4%)	4.358	**0.037**
No	570 (97.3%)	1,427 (98.6%)		
Second-degree relatives with other allergies				
Yes	40 (6.8%)	30 (2.1%)	28.229	**< 0.001**
No	546 (93.2%)	1,414 (97.9%)		
Having pets				
Yes	294 (47.9%)	619 (40.8%)	9.014	**0.003**
No	320 (52.1%)	899 (59.2%)		
History of atopic dermatitis				
Yes	149 (24.3%)	128 (8.4%)	96.970	**< 0.001**
No	465 (75.7%)	1,390 (91.6%)		
History of food allergies				
Yes	91 (14.8%)	63 (4.2%)	74.278	**< 0.001**
No	523 (85.2%)	1,455 (95.8%)		
Frequency of meat consumption				
Per day	401 (66.1%)	889 (60.1%)	6.580	**0.037**
Per week	191 (31.5%)	550 (37.2%)		
Per month	15(2.5%)	41(2.8%)		
Frequency of egg consumption				
Per day	316 (52.1%)	773 (52.3%)	0.006	0.997
Per week	252 (41.6%)	612 (41.4%)		
Per month	38 (6.3%)	93 (6.3%)		
Frequency of dairy consumption				
Per day	337 (55.7%)	818 (55.5%)	0.922	0.631
Per week	222 (36.7%)	559 (37.9%)		
Per month	46 (7.6%)	96 (6.5%)		
Frequency of fruit consumption				
Per day	388 (63.9%)	942 (63.6%)	1.022	0.600
Per week	204 (33.6%)	512 (34.6%)		
Per month	15 (2.5%)	27 (1.8%)		
Frequency of vegetable consumption				
Per day	491 (80.9%)	1,150 (77.7%)	2.685	0.101
Per week	116 (19.1%)	331 (22.3%)		
Per month	0	0		
Frequency of eating high-calorie foods				
Per day	22 (3.6%)	74 (5.0%)	5.899	0.052
Per week	225 (37.3%)	475 (32.2%)		
Per month	357 (59.1%)	925 (62.8%)		
Frequency of soft drink consumption				
Per day	22 (3.6%)	67 (4.6%)	7.814	**0.020**
Per week	244 (40.5%)	501 (34.1%)		
Per month	337 (55.9%)	902 (61.4%)		
Frequency of snack consumption				
Per day	75 (12.4%)	206 (14.0%)	1.231	0.540
Per week	337 (55.6%)	788 (53.4%)		
Per month	194 (32.0%)	481 (32.6%)		

BMI, body mass index. Bold values indicates statistical significance (*P* < 0.05).

### 3.3 Comparison of sleep, emotion, exercise, and screen exposure between children with and without airway allergic diseases

The disease group had significantly higher DSRS-C, OSA-18, and CSHQ scores than those of the control group (all *P* < 0.001). Children with allergic airway diseases are more likely to have sleep disorders and depression than those in the control group. No significant between-group differences were observed in mid-to long-screen time (*P* = 0.168), close-distance screen time (*P* = 0.749), exercise frequency (*P* = 0.544), or daily exercise duration (*P* = 0.652; Table 2).

**Table 2:** Comparison of sleep, emotion, exercise, and screen exposure between the airway allergy group and the control group.

Variables	Airway allergic diseases (*n* = 614)	Control (*n* = 1518)	t/Z	*P* value
**DSRS-C**	7 (5, 10)	6 (4, 9)	−4.835	**< 0.001**
**OSA-18**	0 (0, 2)	0 (0, 1)	−7.834	**< 0.001**
**CSHQ**	46 (42, 51)	44 (41, 48)	−7.210	**< 0.001**
Screen time (mid-/long-distance), min/day	60 (30, 90)	60 (30, 90)	−1.378	0.168
Screen time (close-distance), min/day	60 (30, 90)	60 (30, 90)	−0.319	0.749
Exercise frequency, days/week	4 (2, 5)	4 (2, 5)	−0.607	0.544
Exercise duration, min/day	30 (20, 30)	30 (15, 30)	−0.451	0.652

DSRS-C, depression self-rating scale for children; OSA-18, disease specific quality of life for children with obstructive sleep apnea 18 items survey; CSHQ, children's sleep habits questionnaire. Bold values indicates statistical significance (*P* < 0.05).

### 3.4 Comparison between children with asthma (with or without allergic rhinitis) and those with allergic rhinitis alone

Between-group analyses showed that, compared to children with allergic rhinitis alone, the asthma group had a higher proportion of active smoking (*P* = 0.007), family history of allergy (*P* = 0.006), asthma in first-degree relatives (*P* = 0.016), asthma in second-degree relatives (*P* = 0.032), and self-reported food allergies (*P* = 0.038). Children in the asthma group also had higher CSHQ scores (*P* = 0.031) and longer close-distance screening times (*P* = 0.011). No significant between-group differences were observed for other variables (all *P* > 0.05; Table 3).

**Table 3:** Comparison of all above items between the group with asthma (with or without Allergic Rhinitis) and the group with allergic rhinitis alone.

Variables	Allergic rhinitis (*n* = 381)	Asthma (*n* = 233)	*t*/*Z*/*X*^2^	*P* value
Sex (male), *n* (%)	226 (59.3%)	145 (62.2%)	0.513	0.474
Age	9 (8, 10)	9 (8, 11)	−0.377	0.706
BMI (kg/m^2^)	17.9 (15.6, 22.1)	18.9 (15.8, 22.8)	−1.537	0.124
Screen time (mid-/long-distance), min/day	60 (0, 120)	60 (0, 150)	−1.241	0.214
**Screen time (close-distance), min/day**	60 (12, 108)	60 (0, 150)	−2.552	**0.011**
DSRS-C	7 (2, 12)	7 (1, 13)	−0.531	0.595
OSA-18	0 (0, 2)	0 (0, 2)	−0.188	0.850
**CSHQ**	46 (38, 54)	47 (37, 57)	−2.152	**0.031**
Father's age at birth of child	29 (27, 32)	29 (27, 33)	−0.017	0.987
Mother's age at birth of child	28 (26, 30)	28 (26, 32)	−0.692	0.489
Alcohol consumption				
Never	373 (100.0%)	223 (99.6%)	1.668	0.197
More than once	0 (0%)	1 (0.4%)		
Second-hand smoke exposure				
Never	253 (68.0%)	144 (63.2%)	0.219	0.223
More than once	119 (32.0%)	84 (36.8%)		
Father's highest educational attainment				
Below bachelor's degree	178 (47.6%)	113 (49.3%)	0.174	0.676
Bachelor's degree or above	196 (52.4%)	116 (50.7%)		
Mother's highest educational attainment				
Below bachelor's degree	160 (42.9%)	107 (47.3%)	1.128	0.288
Bachelor's degree or above	213 (57.1%)	119 (52.7%)		
Average annual household income (yuan)				
< 120,000	152 (39.9%)	99 (42.5%)	0.403	0.526
≥120,000	229 (60.1%)	134 (57.5%)		
Feeding methods before 6 months of age				
Breastfeeding	223 (58.8%)	120 (52.6%)	2.232	0.328
Formula feeding	52 (13.7%)	36 (15.8%)		
Combined feeding	104 (27.4%)	72 (31.6%)		
Family history of allergies				
Yes	79 (21.4%)	69 (31.5%)	7.440	**0.006**
No	290 (78.6%)	150 (68.5%)		
First-degree relative with asthma				
Yes	5 (1.4%)	10 (4.6%)	5.799	**0.016**
No	364 (98.6%)	207 (95.4%)		
First-degree relatives with other allergies				
Yes	61 (16.5%)	48 (22.0%)	2.729	0.099
No	308 (83.5%)	170 (78.0%)		
Second-degree relative with asthma				
Yes	6 (1.6%)	10 (4.6%)	4.576	**0.032**
No	363 (98.4%)	207 (95.4%)		
Second-degree relatives with other allergies				
Yes	21 (5.7%)	19 (8.8%)	2.018	0.155
No	348 (94.3%)	198 (91.2%)		
Having pets				
Yes	182 (47.8%)	112 (48.1%)	0.005	0.943
No	199 (52.2%)	121 (51.9%)		
History of atopic dermatitis				
Yes	86 (22.6%)	63 (27.0%)	1.569	0.210
No	295 (77.4%)	170 (73.0%)		
History of food allergies				
Yes	48 (12.5%)	43 (18.5%)	13.360	**0.038**
No	333 (87.4%)	190 (81.5%)		
Frequency of meat consumption				
Per day	244 (64.7%)	157 (68.3%)	0.941	0.625
Per week	124 (32.9%)	67 (29.1%)		
Per month	9 (2.4%)	6 (2.6%)		
Frequency of egg consumption				
Per day	205 (54.5%)	111 (48.3%)	2.287	0.319
Per week	148 (39.4%)	104 (45.2%)		
Per month	23 (6.1%)	15 (6.5%)		
Frequency of dairy consumption				
Per day	202 (53.9%)	135 (58.7%)	1.940	0.379
Per week	141 (37.6%)	81 (35.2%)		
Per month	32 (8.5%)	14(6.1%)		
Frequency of fruit consumption				
Per day	248 (65.8%)	140 (60.9%)	1.707	0.426
Per week	121 (32.1%)	83 (36.1%)		
Per month	8 (2.1%)	7 (3.0%)		
Frequency of vegetable consumption				
Per day	313 (82.8%)	178 (77.7%)	2.376	0.123
Per week	65 (17.2%)	51 (22.3%)		
Per month	0	0		
Frequency of eating high-calorie foods				
Per day	10 (3.2%)	10 (4.4%)	1.428	0.490
Per week	135 (36.0%)	90 (39.3%)		
Per month	228 (60.8%)	129 (56.3%)		
Frequency of soft drink consumption				
Per day	9 (2.4%)	13 (5.7%)	5.265	0.072
Per week	149 (39.6%)	95 (41.9%)		
Per month	218 (58.0%)	119 (52.4%)		
Frequency of snack consumption				
Per day	36 (15.7%)	36 (15.7%)	5.251	0.072
Per week	221 (58.6%)	116 (50.7%)		
Per month	117 (31.0%)	77 (33.6%)		

Bolded values indicate statistical significance (*P* < 0.05).

### 3.5 Logistic regression analysis

**Figure 1:**
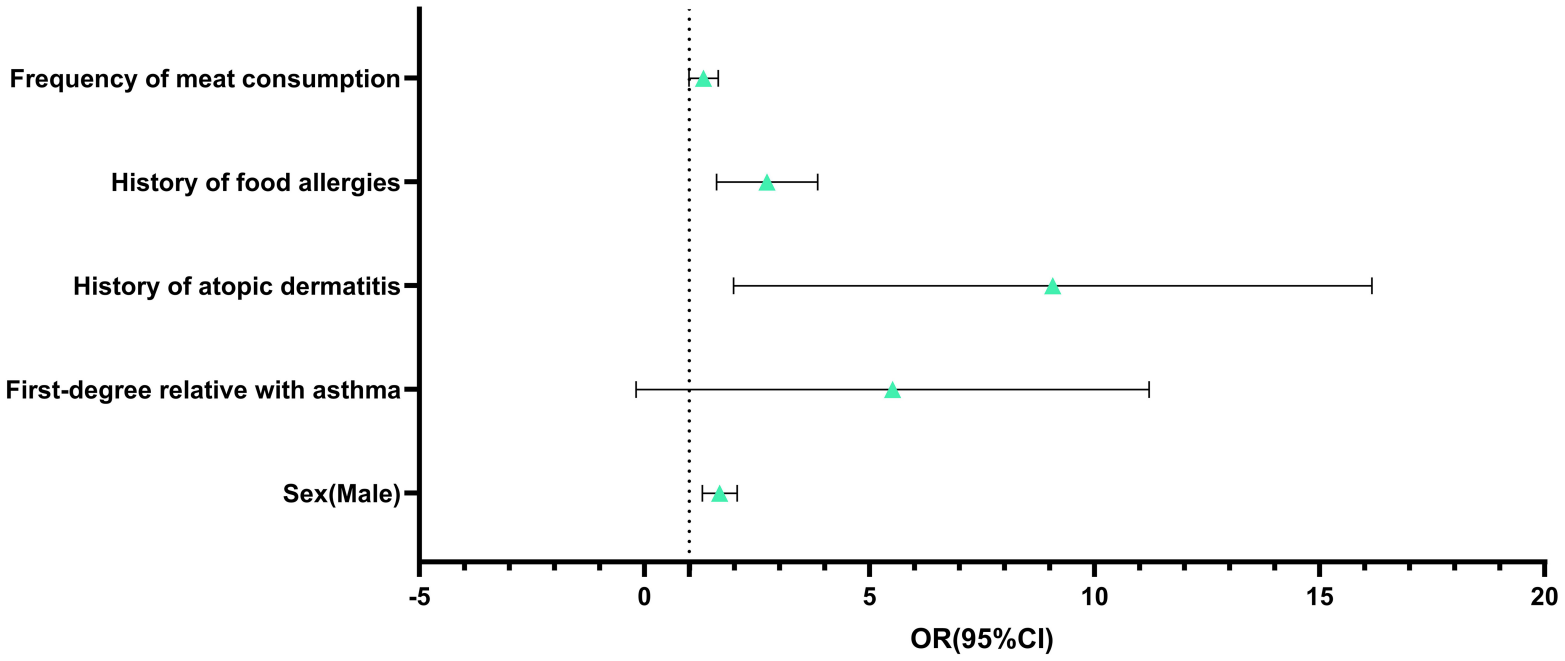
Forest plot for univariate associated factor analysis between the airway allergy group and the control group.

Variables showing statistically significant between-group differences were entered into logistic regression analyses. In the univariate analysis, the results showed that male gender (*P* < 0.001), exposure to secondhand smoke (*P* = 0.030), paternal age at conception (*P* = 0.002), maternal age at conception (*P* = 0.008), paternal higher education (*P* = 0.020), maternal higher education (*P* = 0.011), average annual household income exceeding 120,000 yuan (*P* = 0.007), family history of allergies (*P* < 0.001), history of asthma in first-degree relatives (*P* < 0.001), history of other allergies in first-degree relatives (*P* < 0.001), history of asthma in second-degree relatives (*P* = 0.041), history of other allergies in second-degree relatives (*P* < 0.001), pet ownership (*P* = 0.003), self-reported history of food allergies (*P* < 0.001), self-reported history of atopic dermatitis (*P* < 0.001), higher frequency of meat consumption (*P* = 0.016), and higher frequency of soft drink consumption (*P* = 0.009) were associated with allergic airway diseases. We conducted a multivariate analysis, incorporating variables such as gender, BMI, family history, meat intake, consumption of sugar-sweetened beverages, parental age at childbirth, parental education level, household income, pet ownership, and secondhand smoke exposure into the multivariate model. The results showed that male gender (*P* < 0.001, aOR, 1.644; 95% CI: 1.302 to 2.076), a history of asthma in first-degree relatives (*P* = 0.040, aOR, 3.544; 95% CI: 1.059 to 11.933), a self-reported history of food allergies (*P* < 0.001, aOR, 2.574; 95% CI: 1.691 to 3.916), and a history of atopic dermatitis (*P* < 0.001, aOR, 7.224; 95% CI: 3.087 to 16.903), and frequency of meat consumption (*P* = 0.043, aOR, 1.290; 95% CI: 1.008 to 1.651) were identified as independent associated factors with statistically significant differences (Table 4). Visualizations of the effect sizes and their 95% confidence intervals are shown in Figure 1.

**Table 4:** Associated factor analysis (univariate and multivariable) between the airway allergy group and the control group.

Variables	Univariate		Multivariable	
	OR (95% CI)	*P*-value	OR (95% CI)	*P*-value
BMI	1.018 (0.999, 1.039)	0.071	1.161 (1.015, 1.327)	0.426
**Sex(Male)**	1.491 (1.232, 1.804)	**< 0.001**	1.644 (1.302, 2.076)	**< 0.001**
Father's age at birth of child	0.970 (0.951, 0.989)	**0.002**	0.976 (0.976, 1.011)	0.183
Mother's age at birth of child	0.969 (0.947, 0.992)	**0.008**	0.994 (0.952, 1.011)	0.769
Alcohol consumption	1.218 (0.110, 13.459)	0.872		
**Second-hand smoke exposure**	1.256 (1.022, 1.544)	**0.030**	0.893 (0.695, 1.147)	0.375
**Father's highest educational attainment**	1.251(1.036,1.512)	**0.020**	0.915(0.693,1.207)	0.530
Mother's highest educational attainment	1.279 (1.057, 1.547)	**0.011**	0.909 (0.687, 1.203)	0.604
Average annual household income	1.298 (1.074, 1.569)	**0.007**	0.903 (0.709, 1.150)	0.407
Feeding Methods Before 6 Months of Age	1.091 (0.981, 1.214)	0.107	1.147 (0.882, 1.492)	0.257
Family history of allergies	3.714 (2.853, 4.835)	**< 0.001**	1.420 (0.544, 3.707)	0.474
First-degree relative with asthma	4.191 (1.824, 9.632)	**< 0.001**	3.544(1.059, 11.933)	**0.040**
First-degree relatives with other allergies	3.745 (2.763, 5.075)	**< 0.001**	1.957(0.788, 4.858)	0.148
Second-degree relative with a history of asthma	2.003 (1.030, 3.893)	**0.041**	0.895(0.327,2.451)	0.829
Second-degree relatives with other allergies	3.453 (2.129, 5.600)	**< 0.001**	1.739(0.774,3.908)	0.181
Having pets	1.334 (1.105, 1.611)	**0.003**	1.045 (0.828, 1.318)	0.712
History of atopic dermatitis	3.480 (2.687, 4.506)	**< 0.001**	7.224 (3.087, 16.903)	**< 0.001**
History of food allergies	4.018 (2.871, 5.626)	**< 0.001**	2.574 (1.691, 3.916)	**< 0.001**
Frequency of meat consumption	1.244 (1.041, 1.487)	**0.016**	1.290 (1.008, 1.651)	**0.043**
Frequency of eating high-calorie foods	1.069 (0.909, 1.257)	0.419	0.783 (0.404, 1.519)	0.470
Frequency of soft drink consumption	1.304 (1.070, 1.588)	**0.009**	0.873(0.421, 1.809)	0.715

Bolded values indicate statistical significance (*P* < 0.05).

## 4 Discussion

Allergic airway diseases, primarily allergic rhinitis and asthma, are chronic inflammatory disorders. Allergic rhinitis typically presents with rhinorrhea, sneezing, and nasal obstruction and sometimes with ocular symptoms, whereas asthma is characterized by episodes of chest tightness, wheezing, cough, and reversible airflow limitation (17). In our sample, 28.8% of the children had airway allergic diseases; allergic rhinitis alone accounted for 17.9% had asthma for 10.9%. These estimates are broadly consistent with prior reports, although the prevalence of asthma in our study was higher than that reported in 2010 (3). This difference may reflect a true increase over time, changes in lifestyle and environmental exposure, and improved recognition and diagnostic practices in recent years (18). We relied primarily on parental self-reports without on-site objective confirmation, which may have introduced misclassification and contributed to the higher prevalence estimates (19).

A key finding of this study was that airway allergic diseases are associated with a higher intake frequency of meat and sugar-sweetened beverages, with higher frequency of meat consumption being an independent risk factor for these diseases. Previous research suggests that a Western dietary pattern, characterized by a higher intake of processed foods, refined grains, high-fat dairy products, and added sugars, along with lower consumption of fruits, vegetables, and whole grains, is associated with an increased risk of asthma. This pattern may have pro-inflammatory properties and is linked to higher levels of inflammatory markers such as high-sensitivity C-reactive protein and interleukin-6 (20). Diet may also influence asthma through inflammatory lipid mediators. Western diets are typically richer in ω-6 fatty acids, and arachidonic acid derived from ω-6 fatty acid metabolism can be converted into leukotrienes and prostaglandins. In addition, a lower intake of fruits, vegetables, and whole grains may reduce antioxidant availability. Children with airway allergic diseases often exhibit heightened sensitivity to allergens and other triggers, and inflammatory activation can generate reactive oxygen species (ROS), increasing susceptibility to oxidative stress. Antioxidant deficiency and ROS may activate NF-κB, thereby increasing the secretion of proinflammatory cytokines (21). Emerging evidence also indicates that diet can influence asthma via modulation of gut microbiota and nutrient metabolism. Among children with greater adherence to Western dietary patterns, boys and the overall population have been reported to have a higher likelihood of wheeze symptoms in the past 12 months (22). In our study, although fruit and vegetable intake did not differ substantially between the groups, the meat intake frequency was higher in the disease group, potentially contributing to a more pro-inflammatory dietary profile. In addition, sugar-sweetened beverage consumption has been linked to asthma, and beverages high in free fructose (e.g., high-fructose corn syrup–sweetened drinks) may promote oxidative damage and airway inflammation through advanced glycation end products and RAGE-mediated pathways (23). Mechanistic evidence remains limited and this area warrants further investigation.

Our multivariate analysis identified male sex as the independent factors associated with airway allergic diseases. With respect to sex, boys have a higher prevalence of asthma and rhinitis before puberty, with a shift toward higher prevalence in females after puberty and in adulthood (24–26). These temporal patterns likely reflect the influence of sex hormones, socioeconomic and environmental factors, comorbidities, and differential access to healthcare across settings (27, 28).

Tobacco exposure is an established associated with allergic airway diseases. Among African American and Latino children with asthma, secondhand smoke exposure was associated with an increased risk of exacerbations and poorer asthma control, showing a dose–response relationship even at low exposure levels (29). Tobacco smoke exposure is also associated with a high prevalence of rhinitis symptoms (30). Tobacco smoke contains multiple harmful constituents (e.g., carbon oxides, nitrogen oxides, particulate matter, nitrosamines, polycyclic aromatic hydrocarbons, and carbonyl compounds) that can induce airway inflammation and alter immune function (31, 32).

We also observed associations between airway allergic diseases and socioeconomic/family factors such as parental education level and annual household income. Previous studies have suggested that allergic rhinitis is more prevalent among children and adolescents in high-income countries, whereas severe symptoms are more common in low-income countries (33). Higher-income populations may have greater health literacy and care-seeking behaviors, which could facilitate earlier recognition and diagnosis.

Allergic airway diseases are strongly associated with familial predispositions. A family history of asthma and/or atopic diseases has been linked to persistent wheezing in childhood, reflecting shared environmental exposure and genetic determinants. Genome-wide association studies have demonstrated a substantial overlap in genetic susceptibility loci across allergic diseases. Loci such as IL33, IL1RL1 (IL33R), IL13–RAD50, C11orf30 (EMSY)–LRRC32, and TSLP have been implicated in allergic multimorbidity (34). Multiple genetic variants have been reported in asthma patients. For example, hypomethylation at a CpG site within ALOX12 has been associated with persistent wheezing in children and the HLX1 locus has been linked to childhood asthma symptoms and immune development (35, 36). The interplay between genetic and environmental factors is central to pathophysiology, and further genetic and mechanistic studies are warranted.

Airway allergic diseases are associated with a history of food allergies and atopic dermatitis. Many allergic conditions begin early in life and may follow a characteristic sequence—often referred to as the “atopic march”—in which atopic dermatitis (frequently with food allergy) precedes the later development of allergic rhinitis and asthma (37). Cohort studies indicate that infants with atopic dermatitis in the first 2 years of life have a higher subsequent incidence of allergic rhinitis and asthma at 6–7 years of age; early onset, persistent, and IgE-positive atopic dermatitis appears to confer a particularly high risk (38). Children with food allergies have also been reported to develop allergic rhinitis earlier than those without food allergy (39). Infants sensitized to milk have been shown to exhibit progressively increased airway inflammation and heightened airway responsiveness to histamine (40). Although the underlying mechanisms remain incompletely understood, atopic march provides a useful framework for the prediction, prevention, and early management of allergic diseases.

Additionally, we observed that children in the asthma group reported longer close-distance screen times (e.g., mobile phone use) than those with allergic rhinitis alone. As this was a cross-sectional survey, the directionality of this association cannot be determined; it may reflect behavioral changes secondary to asthma symptoms (e.g., spending more time indoors), unmeasured confounding, or reverse causation. Further longitudinal research is required to confirm this relationship.

This study examined the associated factors for airway allergic diseases among school-aged children in Beijing using a large sample and comprehensive questionnaire battery. This study has several limitations. First, this study did not include objective tests such as pulmonary function tests or skin prick tests, which increases recall bias and may lead to an overestimation or underestimation of the actual prevalence of the disease. Second, cross-sectional study designs do not allow for causal inferences; therefore, it is not possible to establish a causal relationship between the factors associated with allergic airway diseases identified in this study and the diseases themselves. Third, some of the variables in this study are self-reported by parents, which relies on parents' recollection of past events and increases the risk of recall bias. This is particularly critical for variables collected retrospectively, such as the introduction of complementary foods during infancy and exposure to antibiotics. Finally, since all food allergies we recorded were self-reported by parents and no objective tests were conducted, the results cannot distinguish between food sensitization and food allergy. Therefore, we will still need to conduct future studies that combine objective clinical assessments with longitudinal follow-up to validate these findings and clarify causal relationships.

## Data Availability

The original contributions presented in the study are included in the article/Supplementary material, further inquiries can be directed to the corresponding author.
